# Structural Basis for Sequence Specific DNA Binding and Protein Dimerization of HOXA13

**DOI:** 10.1371/journal.pone.0023069

**Published:** 2011-08-01

**Authors:** Yonghong Zhang, Christine A. Larsen, H. Scott Stadler, James B. Ames

**Affiliations:** 1 Department of Chemistry, University of California Davis, Davis, California, United States of America; 2 Department of Molecular and Medical Genetics, Oregon Health and Science University, Portland, Oregon, United States of America; 3 Shriners Hospital for Children Research Department, Portland, Oregon, United States of America; University of South Florida College of Medicine, United States of America

## Abstract

The homeobox gene (HOXA13) codes for a transcription factor protein that binds to AT-rich DNA sequences and controls expression of genes during embryonic morphogenesis. Here we present the NMR structure of HOXA13 homeodomain (A13DBD) bound to an 11-mer DNA duplex. A13DBD forms a dimer that binds to DNA with a dissociation constant of 7.5 nM. The A13DBD/DNA complex has a molar mass of 35 kDa consistent with two molecules of DNA bound at both ends of the A13DBD dimer. A13DBD contains an N-terminal arm (residues 324 – 329) that binds in the DNA minor groove, and a C-terminal helix (residues 362 – 382) that contacts the ATAA nucleotide sequence in the major groove. The N370 side-chain forms hydrogen bonds with the purine base of A5* (base paired with T5). Side-chain methyl groups of V373 form hydrophobic contacts with the pyrimidine methyl groups of T5, T6* and T7*, responsible for recognition of TAA in the DNA core. I366 makes similar methyl contacts with T3* and T4*. Mutants (I366A, N370A and V373G) all have decreased DNA binding and transcriptional activity. Exposed protein residues (R337, K343, and F344) make intermolecular contacts at the protein dimer interface. The mutation F344A weakens protein dimerization and lowers transcriptional activity by 76%. We conclude that the non-conserved residue, V373 is critical for structurally recognizing TAA in the major groove, and that HOXA13 dimerization is required to activate transcription of target genes.

## Introduction

**Figure 1:**
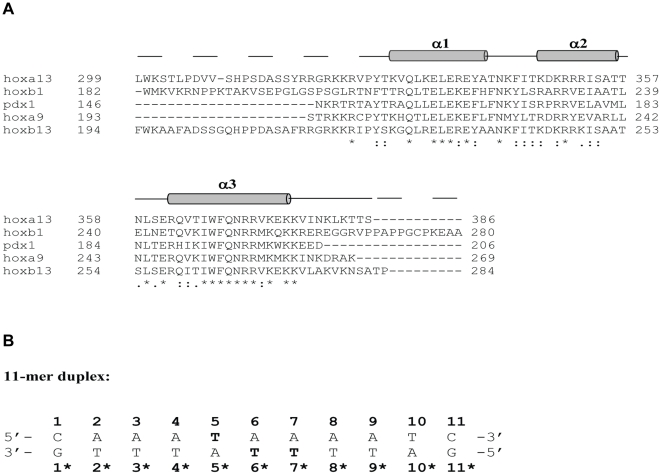
Primary sequence of HoxA13 and 11-mer duplex DNA. (A) Amino acid sequence alignment of HOXA13 with other homeodomain proteins: HOXA9, HOXB13, HOXB1 and Pdx1. Residues at the DNA binding interface are highlighted bold and residues at the dimer interface are colored red. (B) Nucleotide sequence of 11-mer duplex DNA. The sense oligonucleotide strand is numbered from 1 to 11 in the 5′ to 3′ direction and the complementary strand is numbered from 1* to 11* in the 3′ to 5′ direction. The thymine bases that interact with V373 are highlighted bold.

Homeobox (Hox) genes encode a conserved family of transcription factor proteins that are critically important in vertebrate development [1]. In humans, the Hox genes are distributed into four linkage groups (HOXA, B, C, D) comprising 39 genes located on chromosomes 7, 17, 12, and 2 (Fig. 1). Recently, mutations in HOXA13 have been associated with Hand-Foot-Genital-(HFGS) and Guttmacher syndromes (GS), autosomal dominant disorders that profoundly affect limb and genitourinary development causing defects in the digits, carpal/tarsal bones, uterus, bladder, Mullerian ducts, and the external genitalia [2], [3], [4]. In mice, loss of function studies have confirmed a conserved role for HOXA13 in limb and genitourinary development and also identified a novel function for HOXA13 in the developing murine placenta, where it regulates the expression of *Tie2* and *Foxf1a* to facilitate the formation of the placental vascular labyrinth [5], [6], [7].

While it is well-established that HOXA13 is essential for the formation of many different tissues, it is less clear how HOXA13 mediates the tissue-specific expression of its target genes. To address this question, a SELEX approach was used to identify a consensus sequence of 5′-AAATAAAA-3′ preferred by HOXA13 [8]. The core sequence in the major groove (5′-ATAA-3′) is somewhat different from the corresponding sequence (5′-ATCA-3′) recognized by most other HOX proteins [9]. Another unique feature of HOXA13 is that it binds tightly to specific DNA targets (K_d_ = 3.7 nM) without the aid of any cofactors [8]. This is in stark contrast to other known HOX proteins that require MEIS-class [10], [11] and/or PBC-class cofactors [12], [13] to enable high affinity DNA binding. An atomic-resolution structure of HOXA13 bound to duplex DNA is therefore needed to elucidate its sequence-specific DNA binding and provide a structural basis for understanding how mutations in HOXA13 cause HFGS and GS.

Here we report the NMR structure of the murine HOXA13 DNA binding domain (residues 314–386, called A13DBD) bound to an 11-residue DNA duplex. Interestingly, the A13DBD forms a dimer in solution bound to two molecules of duplex DNA, forming a 2:2 complex called, (A13DBD)_2_-(DNA)_2_. The structure contains a positively charged N-terminal arm that forms electrostatic contacts in the minor groove of DNA. Exposed residues on the C-terminal helix make sequence-specific contacts in the major groove. N370 forms critical hydrogen-bonds with the purine base of A5* (astericks indicates base paired with T5, see Fig. 1B for duplex base pair numbering). V373 makes a cooperative network of hydrophobic contacts with pyrimidine methyl groups in the major groove and thus explains the specific recognition of 5′-TAA-3′ in the core sequence. Exposed protein residues, R337 and F344 contact each other at the protein dimer interface. Mutating F344 weakens protein dimerization and diminishes the transcriptional activation function. We propose that homodimerization of HOXA13 might promote multi-valent DNA contacts to help bend the DNA template and recruit the transcriptional machinery needed to regulate target gene expression.

## Results

### NMR-derived Structure of A13DBD

**Figure 2:**
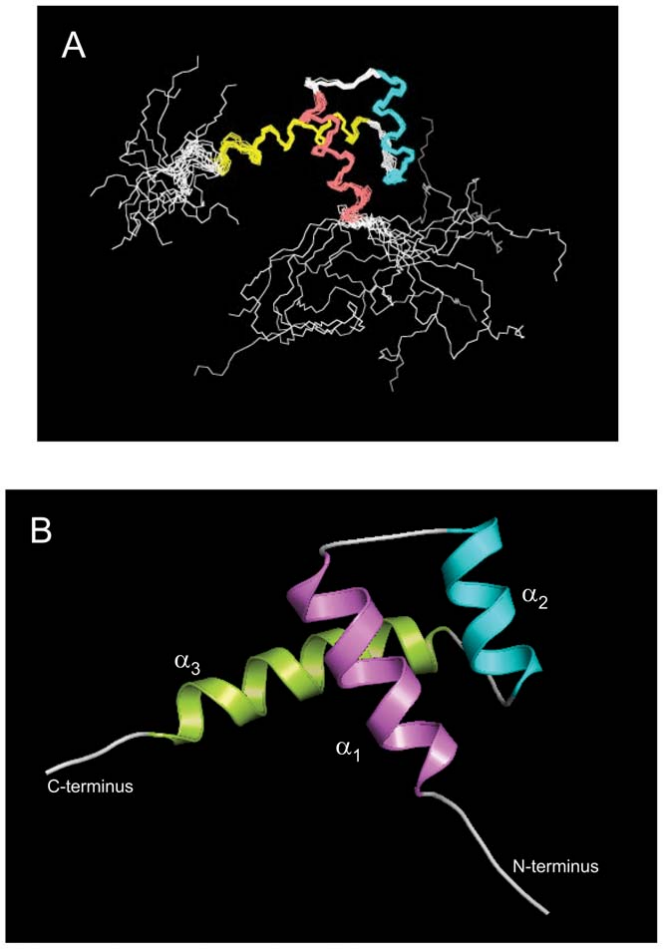
NMR-derived structures of A13DBD in solution. (**A**) Superposition of main chain atoms of 15 lowest energy structures with RMSD of 0.4 Å (main chain atoms). (**B**) Ribbon representation of the energy-minimized average main chain structure. The three α-helices are highlighted magenta (α1, residues 329–341), cyan (α2, residues 347–356), and green (α3, residues 362–379).

The ^1^H-^15^N HSQC NMR spectrum of A13DBD [14] exhibited the expected number of amide resonances with good chemical shift dispersion and uniform intensities, indicative of a folded protein [14]. Sequence specific NMR assignments of A13DBD were analyzed and described previously (BMRB no. 16252). The assigned NMR resonances represent main chain and side chain amide groups that serve as fingerprints of overall conformation. Three-dimensional protein structures derived from the NMR assignments were calculated on the basis of NOE data, chemical shift analysis, and ^3^J_NHα_ spin-spin coupling constants (see Methods). The final NMR-derived structures of A13DBD are illustrated in Fig. 2 (atomic coordinates have been deposited in the RCSB Protein Databank, accession no. 2l7z). Table 1 summarizes the structural statistics calculated for 15 lowest energy conformers with an RMSD of 0.4 Å (main chain atoms) and 1.15 Å for all heavy atoms.

**Table 1:** Structure Statistics for NMR-derived Structures of A13DBD.

NOE restraints (total)	778
intra (i – j = 0)	139
medium (1 ≤ i –j ≤4)	564
long (i – j >4)	75
dihedral angle restraints (*φ* and ψ)	86
hydrogen bond restraints in α–helical regions	54
RMSD from ideal geometry: bond length (Å)	0.0089±0.00014
RMSD from ideal geometry: bond angle (deg)	1.96±0.0097
Ramachandran plot: most favored region (%)	70.1
Ramachandran plot: allowed region (%)	28.3
Ramachandran plot: disallowed region (%)	1.5
RMSD: α-helical regions (main chain) (Å)	0.4±0.089
RMSD: α-helical regions (non-hydrogen) (Å)	1.15±0.11

The main chain structure of A13DBD adopts a canonical homeodomain fold with a positively charged N-terminal arm (residues 321–327) and three α-helices: α1 (residues 329–341), α2 (residues 347–356) and α3 (residues 362–379) shown schematically in Fig. 1A. The first two helices form an antiparallel helical hairpin with electrostatic salt bridges between the two helices at E334/R352 and E338/R350. A long C-terminal helix lays perpendicular to the hairpin helices. Exposed residues on the C-terminal helix (I366, N370, V373, K377, K381) correspond to DNA binding residues in the structures of other homeodomain proteins, Hoxb1 [9], Pdx1 [15], antennapedia [16], [17], engrailed [18], [19], and Hoxa9 [20].

### A13DBD Binding to 11-mer DNA

An 11-mer oligonucleotide (5′-CAAATAAAATC-3′) was the minimal DNA sequence containing the ATAA core [8] that formed a stable duplex and exhibited high affinity and sequence specific binding to A13DBD (see Fig. 1B for duplex base pair numbering). The binding of the 11-mer DNA duplex to A13DBD was quantitatively measured by isothermal titration calorimetry (Fig. S7). The ITC isotherm shows that A13DBD binds exothermically to the duplex (ΔH  =  −30.1 kcal/mol) with an apparent dissociation constant of 7.5 nM and stoichiometric binding ratio of one (Fig. S7). The binding stoichiometry of one was confirmed by NMR titrations that monitored spectral changes of characteristic DNA imino resonances as a function of added A13DBD (Fig. S1).

### Molar Mass of A13DBD/DNA Complex

The molar mass of the A13DBD/DNA complex in solution was determined by measuring its molecular diffusion by NMR. ^15^N NMR relaxation measurements on the A13DBD/DNA complex (R_2_/R_1_ = 35.4) indicate an average rotational correlation time of 18-ns at 37°C, consistent with a spherical mass of 35±4 kDa (Figure S2 and Table 2). NMR pulsed-field gradient diffusion studies [21] determined a translational diffusion coefficient (D = 9×10^−11^ m^2^/s) consistent with a molar mass of ∼35 kDa. Lastly, the molar mass of the A13DBD/DNA complex in solution was determined to be 35±5 kDa based on size-exclusion chromatography (SEC) calibrated using molecular mass standards and multi-angle light scattering (MALS) analysis (Table 2). The observed mass at 35±4 kDa indicates that a dimer of A13DBD (∼20 kDa) must bind to two DNA duplex molecules (∼7 kDa each), giving an expected total mass of 34 kDa. Two 11-mer DNA duplex molecules bound to a protein dimer gives a binding stoichiometry of 2∶2, hereafter designated as (A13DBD)_2_-(DNA)_2_. The dimerization of A13DBD determined above is somewhat inconsistent with previous analytical ultracentrifugation studies that suggest A13DBD is a protein monomer bound to a single DNA hairpin [22]. The A13DBD dimerization may have been disrupted or weakend in the sedimentation studies due to its binding to a DNA hairpin instead of duplex DNA that was used in the current study. The SEC mass analysis measured above as a function of protein concentration indicates a dissociation constant of A13DBD dimerization to be <500 nM, suggesting that (A13DBD)_2_-(DNA)_2_ should be quite stable and remain intact under physiological conditions.

**Table 2:** Molecular Mass of A13DBD (WT and F344A) bound to 11-mer DNA Duplex.

Method	Mass (kDa)
^15^N NMR Relaxation for WT (R_2_/R_1_ = 35.4)	35±4 (τc = 18 ns at 310 K)
^15^N NMR Relaxation for F344A (R_2_/R_1_ = 18.8)	20±3 (τc = 9.8 ns at 310 K)
SEC^1^ for WT	35±5 (at 277 K)
SEC^1^ for F344A	24±4 (at 277 K)
1H NMR gradient-echo^2^	35±5 (D = 9×10-11 m2/s at 310 K)

1 Size-exclusion chromatography (SEC) using Superdex-75 HR/30 column calibrated using molecular mass standards as described by [54].

2 The lateral molecular diffusion coefficient (D) was measured using pulsed-field gradient spin-echo NMR experiments as described by [55].

### Structure of A13DBD bound to duplex DNA

After characterizing the DNA binding properties above, we next set out to determine the NMR structure of (A13DBD)_2_-(DNA)_2_. We have previously reported the NMR spectra and assignments of (A13DBD)_2_-(DNA)_2_ [23]. The backbone chemical shifts, residual dipolar couplings (Fig. S9), and inter-helical NOE patterns (involving L335, I353 and V364) all demonstrate that the overall main chain structure of A13DBD in the complex is quite similar to the structure of the free protein above. Minor structural changes induced by DNA-binding are observed in the N-terminal arm (residues 323–329) and the C-terminal helix in the complex becomes elongated by one helical turn (see Fig. 1A, residues 362–382).

**Figure 3:**
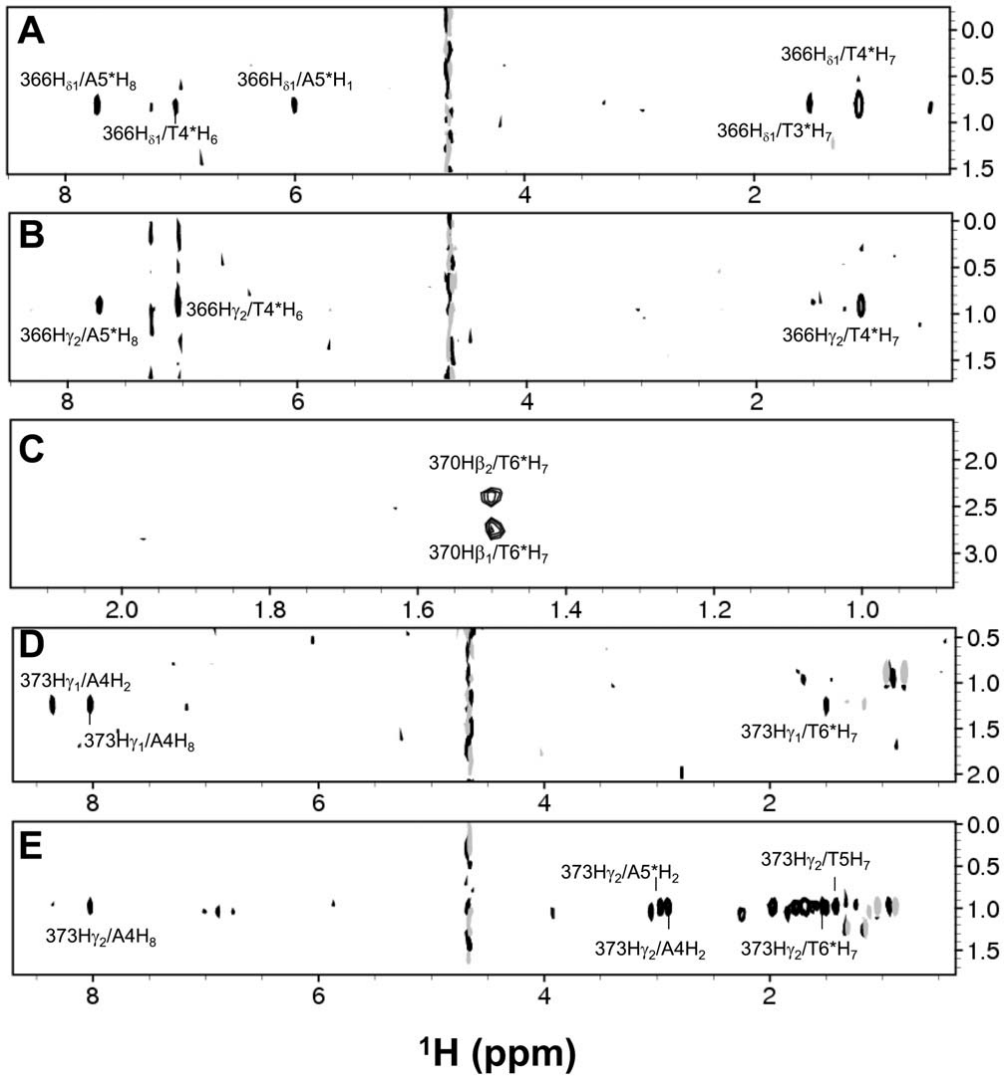
Slices from ^1^H-^1^H planes of a 3D ^13^C-edited (F1) and ^13^C-filtered (F3) NOESY-HSQC spectrum recorded on ^13^C,^15^N-labeled A13DBD bound to unlabeled DNA. Representative two-dimensional planes are shown at different ^13^C chemical shifts and illustrate intermolecular NOEs involving (A) I366 δ1 methyl (F1: ^13^C = 14.4 ppm), (B) I366 γ2 methyl (F1: ^13^C = 19.5 ppm), (C) N370 β methylene (F1: ^13^C = 39.0 ppm), (D) V373 γ1 methyl (F1: ^13^C = 21.0 ppm), and (E) V373 γ2 methyl (F1: ^13^C = 23.0 ppm).

The NMR-derived structures of A13DBD bound to DNA were calculated as described in the Methods using both intermolecular NOEs (Fig. 3) and residual dipolar couplings (Fig. S9). The residues of A13DBD at the DNA interface were first experimentally probed by NMR chemical shift mapping. Chemical shift perturbations due to DNA binding [23] were observed for N-terminal residues (R321, R324 and Y327) and residues in the C-terminal helix (I366, N370, V373, K376 and K377). To identify DNA contacts at atomic resolution, isotope-filtered NOESY experiments (see Methods) were performed on NMR samples that contained ^13^C-labeled A13DBD bound to unlabeled DNA (Fig. 3). The NMR chemical shift assignments for A13DBD in the complex [23] served as the basis for analyzing intermolecular NOE signals from key residues at the protein-DNA interface (Fig. 3). Intermolecular NOEs in the complex were assigned mainly to protein residues (I368, N370 and V373) on the solvent-exposed surface of the C-terminal helix.

The most striking intermolecular NOEs involve protein contacts with pyrimidine methyl groups of thymine residues in the major groove. The 11-mer DNA duplex in this study contains a total of 9 thymines. Each thymine methyl resonance was assigned by generating a series of thymine-to-dexoyuridine (T-to-dU) mutants (T5dU, T6*dU, T7*dU and T4*dU) in which the pyrimidine methyl group for each thymine residue was replaced with a hydrogen atom (see Fig. 1B for duplex base pair numbering). Analysis of 2D ^13^C-filtered (F1 and F2) NOESY spectra that specifically probed unlabeled DNA in these mutants (Fig. S8) provided assignments for pyrimidine methyl groups and H6 resonances from all nine thymines, and H8 resonances in adjacent adenines. The pyrimidine methyl resonances of T5, T6* and T7* interact closely with both side-chain methyl groups of V373, forming an intricate network of hydrophobic contacts (Fig. 3). The T6* methyl is also close to the β-methylene group of N370. T3* and T4* methyl groups interact closely with both methyl groups of I366. Thus, the observed intermolecular NOEs (Fig. 3) represent mostly hydrophobic interactions in the major groove and give rise to a total of 38 intermolecular NOEs used in the structure calculation described in the Methods.

**Figure 4:**
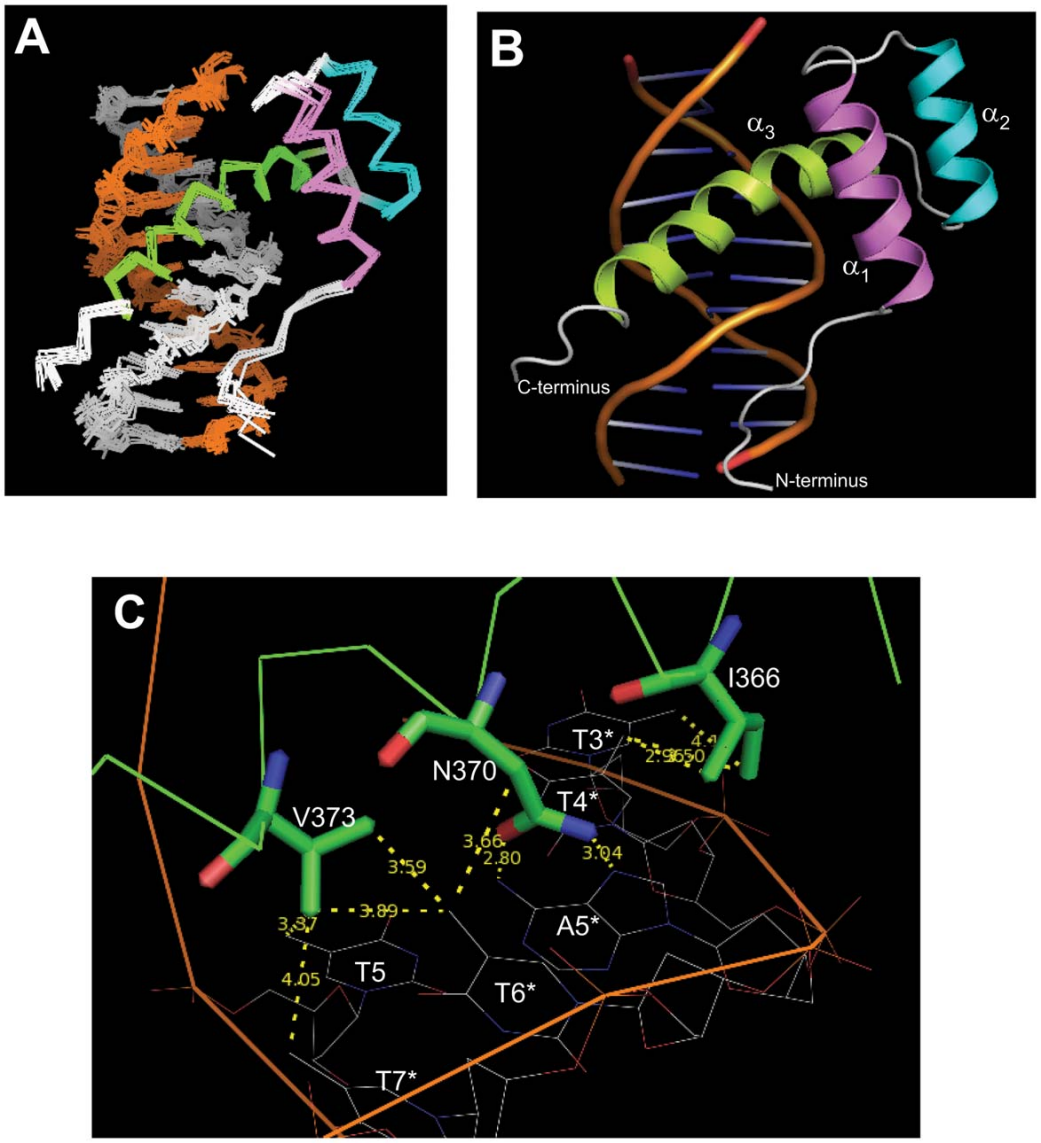
NMR-derived structures of A13DBD bound to 11-mer DNA duplex. (A) Ensemble of 10 lowest energy structures with RMSD of 0.67 Å for A13DBD and 0.69 Å for DNA. The helices are colored as defined in Fig. 2. (B) Ribbon diagram of energy-minimized average structure of A13DBD/DNA complex. The C-terminal helix (green) of the protein contacts the major groove in duplex DNA, and the flexible N-terminal arm interacts with the minor groove. (C) Close-up of the binding interface in the major groove. The key residues are shown as sticks (green) and wires (yellow). The two methyl groups of V373 are close to the methyl groups of T5, T6* and T7* of DNA, the O atom in N370 side-chain is hydrogen-bonded to the amino group of A5*, and the two methyl groups of I366 are close to the pyrimidine methyl groups of T3* and T4*.

The final NMR-derived structures of A13DBD bound to duplex DNA are shown in Fig. 4 (RCSB Protein Databank accession no. 2l5d; see movie S1 and structural statistics in Table 3). The ensemble of structures consistent with the NMR data is shown in Fig. 4A (RMSD  = 0.67 Å). The energy minimized average structure is shown as a ribbon diagram in Fig. 4B. The N-terminal arm region (residues 324–329) of A13DBD makes important electrostatic contacts in the minor groove of DNA. Positively charged protein residues K322 and K323 form electrostatic contacts with backbone phosphate groups of T8*, T10, and C11. The R324 side-chain forms hydrogen bonds with the purine base of A8 and electrostatic contacts with the T6* backbone. The phenolic group of Y327 forms a hydrogen-bond with the backbone phosphate of A5*. The exposed residues of the C-terminal helix (residues I366, N370 and V373) of A13DBD all make important sequence-specific contacts in the major groove (Fig. 4C). The side-chain of N370 forms contacts with the pyrimidine methyl of T6* and hydrogen-bonding contacts with the purine base of A5*: The side-chain carbonyl oxygen and amide proton of N370 are hydrogen-bonded to the amine group and N7 atom of A5*, respectively. Also noteworthy are the network of hydrophobic contacts between the side-chain methyl groups of V373 and pyrimidine methyl groups of T5, T6* and T7*. Additional hydrophobic contacts are formed by the side-chain methyl groups of I366 and pyrimidine methyl groups of T3* and T4*, and H8 atom of A5*. Positively charged residues (R371, R372 and K381) at the end of the C-terminal helix form electrostatic contacts with the backbone phosphate group of A4, T7* and T8*.

**Table 3:** Structure Statistics for NMR-derived Structures of A13DBD/DNA Complex.

Intermolecular NOEs	38
^1^D_HN_ RDC	25
RDC Q-factor	0.078
Ramachandran plot: most favored region (%)	84.4
Ramachandran plot: allowed region (%)	14.1
Ramachandran plot: disallowed region (%)	1.5
RMSD (Å): Protein backbone atoms	0.67
RMSD (Å): DNA heavy atoms	0.69
RMSD (Å): Protein backbone + DNA heavy atoms	0.68

### Mutagenesis of DNA Binding Site

To experimentally verify the sequence-specific DNA contacts suggested by our structure (Fig. 4C), the following protein mutants (I366A, N370A, and V373G) were constructed and characterized for DNA binding and transcriptional activity. All of the mutants are stably folded (see DSC data in Fig. S3) and retain the same NMR spectrum and structure as the wildtype protein (Fig. S4). DNA binding to each of the mutants was monitored by ITC (Fig. S7 and Table 4). The N370A mutant binds to DNA with ∼1000-fold lower affinity compared to wildtype, whereas I366A and V373G each bind to DNA with 3-4-fold lower affinity. The corresponding mutants in the full-length HOXA13 protein showed reduced activity in transcriptional reporter assays. The N370A mutant showed a complete loss of reporter activity, whereas I366A and V373G showed a 40 and 58 percent reduction in reporter activity respectively (Fig. S5). These results confirm that I366, N370 and V373 all participate in DNA binding and have important biological effects on transcription.

**Table 4:** ITC parameters for DNA binding by A13DBD and mutants.

A13DBD	WT	WT	WT	WT	I366A	N370A	V373A	V373G	V373G	V373G
DNA	WT	T5U	T5C	T5UT6*U	WT	WT	WT	WT	T5U	T5C
K_d_ (nM)	7.5	14.5	285.7	40.3	43.7	862.1	18.7	137.2	236.0	617.3
ΔH(kcal/mol)	-30.1	-24.3	-38.3	-23.3	-21.8	-0.01	-23.0	-1.6	-12.9	-25.1

T5U: T-to-dU DNA mutant (deoxythymine mutated to deoxyuridine).

T5C: T-to-C DNA mutant at 5 position of Strand 1 (Strand 1: 5′-CAAACAAAATC-3′, Strand 2: 5′-GATTTTATTTG-3′).

T5UT6*U: T-to-dU DNA mutant (deoxythymine mutated to deoxyuridine).

### DNA Binding Affinity and Sequence Specificity

The V373G mutation was further analyzed for its affinity to bind various DNA sequences. Fluorescence polarization anisotropy assays were use to calculate the K_d_ values of wt and V373G A13DBD with different DNA sequences (Table 5). When compared to wildtype A13DBD, V373G exhibited greater than 10-fold less affinity for all DNA sequences, suggesting that V373 in A13DBD is important for DNA binding.

**Table 5:** K_d_ values of A13DBD wt and V373G measured by fluorescence depolarization.

DNA Sequence	wt K_d_ (nM)	V373G K_d_ (nM)
wt	2.19±0.06	56.6±2.25
T5C	49.2±4.97	615.8±56.1
T5U	4.01±0.28	55.0±4.35
T6*C	5.95±0.64	79.3±6.93
T6*U	16.4±2.07	200.4±10.9

Sequence-specific DNA binding was examined by changing T5 and T6* nucleotides and comparing affinity for the different binding sites with wt A13DBD. A thymine-to-deoxyuridine change at T5 in the A13 binding site resulted in a 2-fold change in affinity, however when changed to a cytosine the affinity decreased by approximately 20-fold. Similarly a thymine-to-deoxyuridine and thymine-to-cytosine change at T6* resulted in a 3-fold and 7-fold change in affinity, respectively. This suggests that both the methyl at position 5 and the oxygen at position 6 in T5 and T6* are important for DNA sequence recognition by A13.

**Figure 5:**
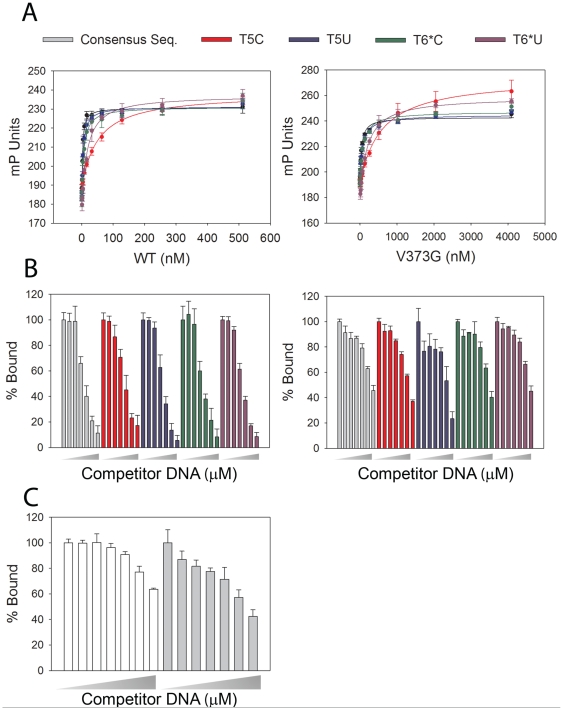
Effects of site specific changes in the A13DBD DNA binding site and mutation of V373 on DNA binding affinity. Individual mutations to the HOXA13 DNA binding site (Table S1) are depicted by each line or bar color. (**A**) Overlay plots show binding of wt A13DBD (left panel) or V373G A13DBD (right panel) to various DNA mutants. (**B**) Competitive displacement assays, for wt (left panel) and V373G (right panel). Wedges indicate an increasing amount of competitor DNA at concentrations of 0, 0.05, 0.1, 0.5, 1, 2.5, and 5 µM. (**C**) Displacement of the high affinity consensus sequence by a scrambled oligonucleotide on wt (white bars) vs. V373G (gray bars). Wedges indicate an increasing amount of srcambled competitor DNA at concentrations of 0, 0.05, 0.1, 0.5, 1, 2.5, and 5 µM. All data represent the average of three independent measurements for each protein and oligonucleotide concentration. Error bars represent the standard deviation of the three independent experiments.

Competitive displacement experiments were performed to assess the sequence specificity of DNA binding to wt and V373G A13DBD (Fig. 5A). Labeled oligonucleotide was bound and competed away from each protein by adding various amounts of a corresponding non-labeled oligonucleotide (Fig. 5B). For all DNA sequences, wt A13DBD was displaced more effectively when compared to V373G, as expected from their large differences in K_d_. The relative specificity of the protein-DNA interaction for wt versus V373G HOXA13 was inferred by comparing how much scrambled oligonucleotide was needed to displace binding of the consensus DNA sequence (Fig. 5C). Wt HOXA13 bound the consensus DNA binding site strongly, and 1 µM or greater of scrambled oligonucleotide was required to compete HOXA13 away. The V373G mutant protein bound much less tightly to the consensus binding site and as little as 50 nM scrambled oligonucleotide demonstrated the ability to compete for binding.

### Protein Dimerization of A13DBD

The structure of the protein dimer in the (A13DBD)_2_-(DNA)_2_ complex could not be directly probed in our NMR experiments due to spectral symmetry and an apparent lack of intermolecular NOEs at the dimer interface, perhaps due to the dimerization off-rate being faster than the NOE (T1) time scale. The NMR spectral symmetry causes our experiments to view only half of the dimer complex (i.e. one A13DBD molecule bound to one DNA duplex). Therefore, we developed an alternative approach to infer the structure of the protein interface by using a site-specific mutagenesis analysis. Inspection of the NMR structure of A13DBD bound to DNA reveals a relatively small exposed surface (solvent exposed surface area  = 422 Å^2^) that would be capable of forming a dimerization binding site. This exposed surface contains a hydrophobic residue (F344) that very likely might form a hydrophobic contact at the dimer interface. To test this hypothesis, we made the mutant F344A that remains structurally folded and does not affect DNA binding (Fig. S7). As expected, the F344A mutant dramatically weakens the dimerization binding interaction as evidenced by SEC/MALS data, indicating an apparent molar mass of 25±4 kDa for the F344A complex compared to 35±5 kDa for that of wildtype (Fig. S6). This result indicates that the F344A mutation weakens the protein dimerization affinity (by at least 10-fold), consistent with F344 being located in the dimerization binding site. We next surveyed the available crystal structures of homedomains and found that PDX1 forms a homodimer [15]. Interestingly, the dimerization site on PDX1 contains an exposed tyrosine (corresponding to F344 in HOXA13) that interacts closely with a conserved arginine (corresponding to R337 in HOXA13). This pair of residues was then mutated in A13DBD (R337A/F344A) and tested for their effect on protein dimerization. The double mutant does not affect DNA binding, but shows a pronounced weakening of the dimerization affinity compared to that of F344A (Fig. S6). Lastly, an exposed lysine (K343) next to F344 was mutated (K343E), and this charge reversing mutation remained structurally intact but lowered the dimerization affinity by ∼5-fold. In short, exposed residues (R337, K343 and F344) all contribute to the dimerization binding energy and must be located at the dimerization binding site.

**Figure 6:**
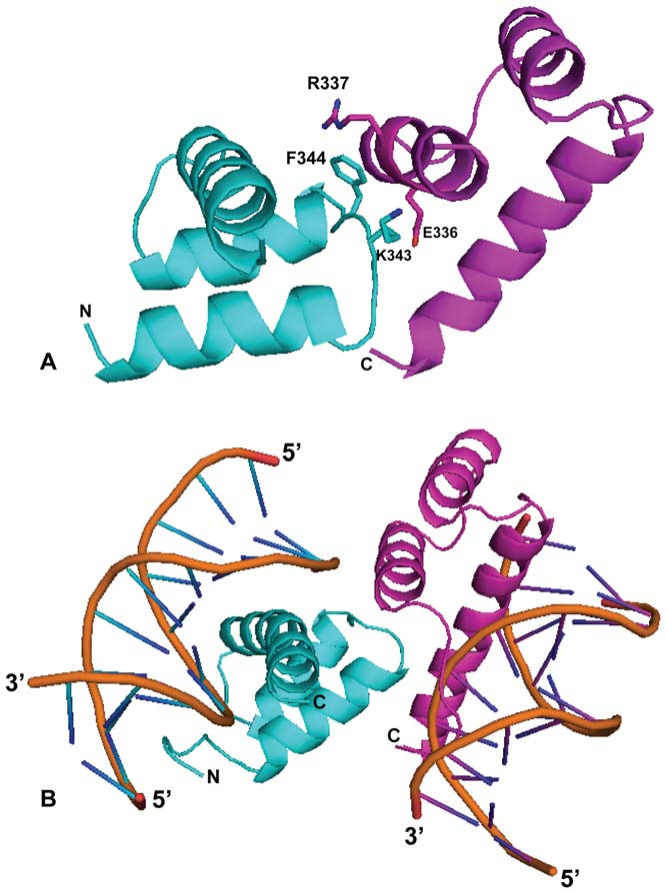
A13DBD protein dimerization. (A) Main chain structure of A13DBD dimer calculated by HADDOCK. Key residues at the dimer interface (R337, K343 and F344) are highlighted. Mutating R337, K343, and/or F344 dramatically weakens the dimerization affinity. (B) Structure A13DBD dimer bound to two DNA duplex molecules.

These dimerization site residues (R337, K343 and F344) were then used as unambiguous restraints to calculate the structure of the dimer using HADDOCK (see methods). The calculated structure of the dimer is very similar to the structure of a protein dimer predicted by homology modeling based on the PDX1 crystal structure [15]. The structural model of the A13DBD dimer is shown in Fig. 6A. In this dimeric structural arrangement, the two duplex DNA molecules are bound at opposite ends of the dimer (Fig. 6B). The structure of the A13DBD dimer therefore has two exposed DNA binding sites that we suggest may bind to multiple sites on the same DNA duplex or perhaps bind to two separate DNA strands.

### Functional Role of A13DBD Dimerization

**Figure 7:**
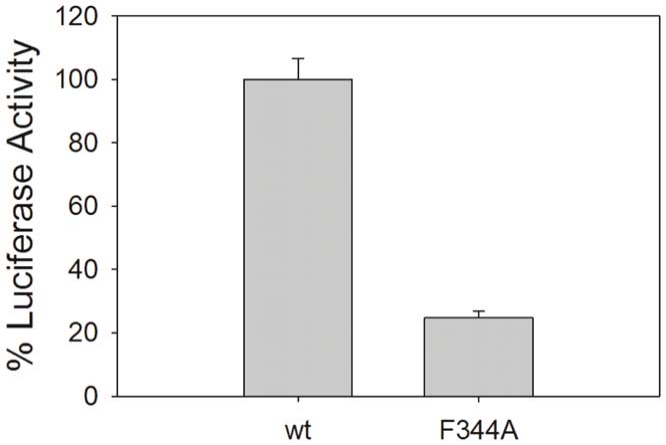
Luciferase assay in NG108-15 cells using a HOXA13 wt and F344A mutant. Percent luciferase activities (relative to wt control) are indicated on the *y* axis. HOXA13 wt or F344A (normalized to renilla and empty vector controls) are plotted on the *x* axis. Results are the mean percent values calculated from three independent experiments. Error bars represent the standard deviation of the three independent experiments.

Recognizing that the (A13DBD)_2_-(DNA)_2_ complex was highly stable during its purification and NMR analysis, we hypothesized that protein dimerization in this complex must exist under physiological conditions and may be required to regulate target gene expression. To test the functional role of protein dimerization, wild type HOXA13 protein and mutant HOXA13 protein bearing a substitution at the F344 dimerization site (F344A) were evaluated for their capacity to regulate gene expression from the *EphA7* enhancer element that we previously identified to be regulated by HOXA13 *in vitro* and was bound by full length HOXA13 in embryonic tissues [22]. Analysis of normalized luciferase expression confirmed that wt HOXA13 regulates gene expression through the *EphA7 cis*-regulatory DNA element (Figure 7). In contrast, substitution of the dimer-forming phenylalanine residue with alanine at the position 344 generated a mutant (F344A) that consistently reduced luciferase expression from the same *EphA7 cis*-regulatory element by greater than seventy five percent, confirming the importance of dimerization and F344 in target gene regulation (Figure 7).

## Discussion

In this study, we present the NMR structure of the A13DBD dimer bound to duplex DNA (Fig. 4) and establish that HOXA13 dimerization is functionally important for activating transcription (Fig. 7). The A13DBD dimer binds symmetrically to two separate DNA duplex molecules at both ends of the dimer (Fig. 6B). At each DNA binding site, the N-terminal arm of A13DBD (residues 324–329) makes electrostatic contacts in the DNA minor groove (Fig. 4B), and a mutation in this region (R326G) is implicated in Hand-Foot-Genital syndrome [24]. The solvent exposed C-terminal helix (I366, N370 and V373) makes sequence specific contacts in the major groove (Fig. 4C), and mutations in the C-terminal helix are linked to HFGS [3]. The N-terminal arm and last four residues of the C-terminal helix are structurally disordered in the absence of DNA (Fig. 2A). These disordered residues adopt a rigid structure upon binding to DNA (Fig. 4A). We suggest the dynamic disorder at the N- and C-termini may facilitate an induced-fit mechanism of DNA target recognition to help explain the very high DNA binding affinity and sequence specificity. Another distinctive structural feature of A13DBD (not seen in other known homeodomains) is that exposed hydrophobic residues (I366 and V373) form an extensive network of hydrophobic contacts with thymine methyl groups in the major groove. Particularly striking are the cooperative hydrophobic contacts between V373 and the methyl groups of T5, T6* and T7* that explain the highly specific recognition of 5′-TAA-3′ in the major groove. In most other homeodomains, V373 is replaced with methionine that specifically contacts cytosine (instead of thymine) in the major groove as seen in the crystal structures of HOXB1 [9] and other known homeodomains [15], [16], [17], [19], [25]. This valine substitution may explain in part why cytosine is *NOT* present in the core DNA sequence for HOXA13 (5′-AATAA-3′ [8]).

HOXA13 belongs to the Group 13 subclass of the *abdominal B* hox genes which in humans and mice contain four conserved members (*Hoxa13*, *Hoxb13*, *Hoxc13* and *Hoxd13*) that share about 90% sequence identity in their homeodomain regions. As a result, their structures and DNA sequence recognition should all be similar. Indeed, V373 (that is critical for sequence-specific DNA binding) remains invariant in all members of this Group 13-subfamily, but is not conserved in many other well known homeodomain proteins like HOXB1 PDX1, ENGRAILED, and HOXA9 (Fig. 1A). Our structure of A13DBD reveals that the two side-chain methyl groups of V373 are responsible for the recognition of 5′-TAA-3′ in the major groove due to the network of hydrophobic contacts it makes with pyrimidine methyl groups of T5, T6* and T7* (Fig. 4C). By contrast, a methionine substituted in place of valine at this position in HOXB1 makes very different contacts in the major groove [9]. We suggest that all members of the Group 13-subfamily (HOXA13-D13) may recognize a similar 5′-TAA-3′ sequence element, because they all contain valine instead of methionine at this key position. The Group 13-subclass is unique in having valine conserved at this position, while all other homeodomains are known to have either methionine or alanine. Previous analysis of the full-length consensus HOXA13 binding site indicates that DNA affinity and specificity may also be influenced by the nucleotides flanking the 5′-TAA-3′ core sequences, which could explain how each of the Group 13 homeodomain proteins can regulate different developmental processes even in the same tissues. Indeed, while HOXA13 and HOXD13, are strongly co-expressed in the limb during development, their loss of function phenotypes are remarkably different, suggesting that each protein must regulate a unique cohort of genes in the developing limb [6], [26], [27].

**Figure 8:**
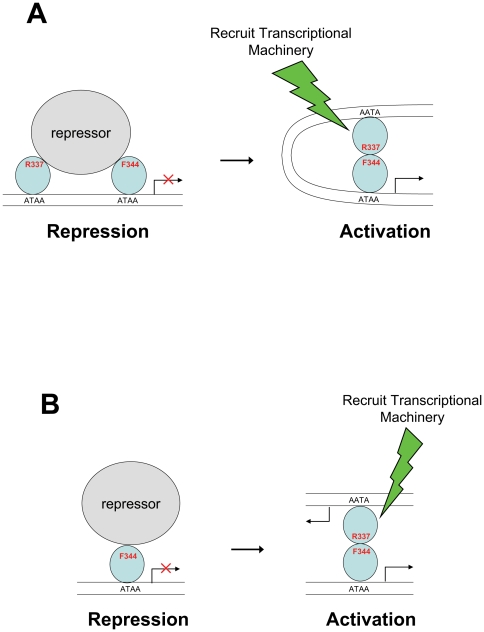
Schematic models of transcriptional regulation by HOXA13. HOXA13 (colored light blue) represses transcription when it is monomeric (left) and activates transcription when it forms a homodimer (right). The HOXA13 homodimer can bind to two DNA sites (ATAA) within the same DNA strand (A) or on two separate DNA molecules (B). The HOXA13 homodimerization activates transcription by bending the DNA that may help to recruit the transcriptional machinery necessary to activate gene expression. Monomeric HOXA13 inhibits transcription (left) under conditions that promote dimer dissociation (e.g. binding to a repressor molecule that blocks the dimerization site marked by R337 and F344).

Our structural analysis reveals that A13DBD forms a protein dimer in solution (Fig. 6), which is functionally important for the regulation of HOXA13 target gene expression (Figs. 7–8). Mutations that weaken HOXA13 dimerization (F344A and R337A/F344A) also diminish the amount of transcriptional activation (Fig. 7). Hence, the HOXA13 dimerization suggests a mechanism to explain how HOXA13 can both activate and repress target gene expression [28]. We propose that the HOXA13 dimer can form multivalent contacts with two separate sites on the same DNA strand (Fig. 8A). The dimerization of HOXA13 in this context would cause a bending of duplex DNA (Fig. 8A), which has been previously shown to affect transcription [29]. Alternatively, the HOXA13 dimerization might connect two separate DNA duplexes (Fig. 8B) and thus simultaneously activate transcription at both sites. Conditions that induce dimerization of HOXA13 (e.g. absence of a repressor molecule or high concentration of HOXA13) are expected to activate transcription (Fig. 8, right panel). Conversely, conditions that promote dissociation of the HOXA13 dimer (e.g. binding to a repressor protein) are predicted to inhibit transcription (Fig. 8, left panel).

The HOXA13 dimerization may also have a role in explaining possible disease phenotypes linked to polyalanine expansion mutants [30], [31], [32]. Wild-type HOXA13 and HOXD13 have been suggested to be mislocalized when coexpressed with mutant HOXA13 that contains polyalanine expansions in vitro [32]. The presence of additional alanine residues in the polyalanine tracts of HOXA13 have been suggested to cause protein aggregation [33]. If the polyalanine mutant of HOXA13 forms a dimer with wildtype HOXD13, then this heterodimerization might sequester HOXD13 in the cytoplasm, which could result in lower levels of normal HOXD13 in the nucleus and cause disease [34]. However, studies on transgenic mice suggest that HOXA13 polyalanine tract mutants are rapidly degraded in the cytosol and may not colocalize with HOXD13 [30]. More rigorous biophysical analysis and co-localization studies are needed to directly probe a binding interaction between HOXA13 and HOXD13, and characterize any consequent sequestration by polyalanine tract mutants.

The R337A/F344A dimerization sites in HOXA13 might also interact with co-factor proteins. Indeed co-factors such as MEIS- and PBC-class proteins have been shown to interact with HOX proteins, which promote DNA binding and regulate target gene expression [10], [11], [12], [13], [35]. However, the typical interaction motifs for the PBC-class co-factors are not apparent in HOXA13 [35], [36]. It is conceivable that a cofactor protein might interact with F344 and/or R337 at the dimerization site, which could modulate the dimerization equilibrium of HOXA13 and/or help recruit transcriptional machinery needed to regulate target gene expression (Fig. 8, left). An important next step will be to probe the interaction of cofactor proteins with the exposed residues at the dimerization site (Fig. 6).

An important distinguishing feature of HOXA13 is its capacity to bind and bend DNA in the absence of any co-factors. We suggest that homodimerization of HOXA13 by itself may be sufficient to regulate target gene expression. This self-sufficient activation by HOXA13 should effectively increase the number of tissues where HOXA13 can regulate target gene expression and could explain why HOXA13 controls so many specific developmental processes. A key question moving forward is to determine what cellular conditions will promote and/or affect the HOXA13 dimerization. One possibility is that a repressor protein may compete for binding at the HOXA13 dimerization site and prevent dimerization as depicted in Fig. 8. Also, the dimerization equilibrium might be highly reversible and controlled thermodynamically by mass action or other changes in the cell. Future studies are needed to identify specific binding partner proteins and understand how the regulatory domain in HOXA13 might be involved in modulating dimerization to control gene expression.

## Materials and Methods

### HOXA13 reference sequence

Amino acid sequence numbering was defined using the *Mus musculus* HOXA13 protein sequence from GenBank Accession number:AAB03322.1 (NCBI) (see Fig. 1).

### Protein Expression and Purification

HOXA13 DNA-binding domain (G320-S386) was subcloned into the XhoI and BamHI restriction sites in the pET-15b expression vector to make pHisA13DBD plasmid. All site-directed mutation constructs were generated by using the QuikChange mutagenesis kit (Stratagene), and the presence of these mutations was confirmed by DNA sequencing. The plasmids were transformed into BL21(DE3) competent cells for protein over-expression. Un-labeled proteins were over-expressed in LB medium, and purified by following the standard His-tag protein purification protocol. The pure His-tagged proteins were cleaved by thrombin to remove His-tag, and then purified by size-exclusion chromatography (Superdex-75). Uniformly ^15^N-labeled and ^13^C/^15^N-labeled were over-expressed in M9 with ^15^NHCl and ^15^NH_4_Cl/^13^C-glucose by using high-yield protein expression protocol [37], and purified as described previously [14]. The identity and integrity of the final protein sample was confirmed by SDS-PAGE.

### Preparation of Protein•DNA Complex

Single-stranded oligonucleotides (*5′-CAAATAAAATC-3′* and *5′-GATTTTATTTG-3′*) and T-to-dU mutant oligonucleotides with HPLC purification were obtained from http://us.bioneer.com/, Inc. For DNA duplex preparation, two single stranded DNA oligonucleotides in annealing buffer (10 mM Tris-HCl, 100 mM NaCl, 0.1 mM EDTA, pH 7.0) were mixed in 1∶1 molar ratio, and heated to 95°C for 5 min, then cooled to room temperature over the course of several hours. Concentrations were measured and calculated by absorbance at 260 nm. To make protein•DNA complex, protein was mixed with duplex DNA at 1∶1 molar equivalent amount, and the mixture was incubated at 15°C for one hour. Finally the protein-DNA complex was purified by gel filtration chromatography (Superdex-75) to remove any unbound DNA.

### Molecular Mass Analysis

Size exclusion chromatography (SEC) was performed on a Superdex 75 HR 10/30 column (GE Healthcare) at 4°C. A 0.1 ml aliquot of protein was loaded onto the column and eluted at a flow rate of 0.5 ml/min. Molecular masses were analyzed by analytical SEC performed in-line with a multi-angle light-scattering (MALS) miniDawn instrument with a 690-nm laser (Wyatt Technologies, Inc.) coupled to refractive index instrument (Optilab Rex, Wyatt Technologies, Inc.). The molar mass of chromatographed protein was calculated from the observed light scattering intensity and differential refractive index [38] using ASTRA software (Wyatt Technologies, Inc.) based on Zimm plot analysis using a refractive index increment, dn/dc = 0.185 L g^-1^ [39]. Apparent molecular weights were also calculated using a standard curve of Ve/Vo versus the log of the molecular weights of standard proteins: β-amylase (200 kDa), alcohol dehydrogenase (150 kDa), transferrin (81 kDa), carbonic anhydrase (29 kDa), and myoglobin (17 kDa). Vo is a void volume obtained using blue dextrane (2000 kDa) and Ve is the elution volume.

### NMR Spectroscopy

A13DBD (unlabeled, ^15^N- or ^15^N/^13^C-labeled) was exchanged into NMR buffer (20 mM NaPO_4_, 10%D_2_O, pH 7.0) and concentrated to about 200–700 µM. For A13DBD•DNA complex, the combined fractions from gel filtration chromatography (Superdex-75) containing pure A13DBD•DNA was exchanged to NMR buffer (20 mM NaPO_4_, 5 mM MgCl_2_, 10%D_2_O, pH 6.0) and concentrated to about 0.5 mM. All NMR experiments were performed at 285K for A13DBD samples and at 310K for A13DBD•DNA complex samples, on Bruker Avance III 600 or 800 MHz spectrometers equipped with a four-channel interface and triple-resonance cryoprobe (TCI) with pulse field gradients. The ^15^N-^1^H HSQC spectra were recorded on a sample of ^15^N-labeled free A13DBD and a sample of ^15^N-protein labeled A13DBD•DNA complex. The 3D experiments for NMR resonance assignments of free protein and protein/DNA complex were described previously [14], [23]. Stereospecific assignments of chiral methyl groups of valine and leucine were obtained by analyzing ^1^H-^13^C HSQC experiments performed on a sample that contained 10% ^13^C labeling of A13DBD [40]. NOE distance restraints for the A13DBD structure calculation were obtained from the analysis of 3D NOESY experiments including ^15^N-edited NOESY-HSQC and ^13^C-edited NOESY-HSQC recorded at 800 MHz on ^15^N-labeled A13DBD and ^13^C/^15^N-labeled A13DBD samples. For A13DBD/DNA complex, a ^13^C/^15^N-labeled A13DBD bound to unlabeled DNA was used to record 3D ^13^C-edited NOESY-HSQC to probe all NOEs within the labeled protein. 3-D ^13^C/^15^N-filtered-(F3) and ^13^C-edited (F1) HSQC-NOESY [41] selectively probed intermolecular NOEs, and 2D ^13^C/^15^N-double-filtered NOESY selectively probed DNA intramolecular NOEs. The NOE mixing time was 120 ms in all NOESY experiments. NMR data were processed using NMRPipe [42] and analyzed with SPARKY.

### NMR Residual Dipolar Coupling Measurements

For the measurement of residual dipolar couplings (RDCs) of A13DBD bound to duplex DNA, the filamentous bacteriophage Pf1 (Asla Biotech Ltd, Latvia) was used as an orienting medium. Pf1 (10 mg/ml) was added to the ^15^N-labeled A13DBD protein/DNA complex sample at pH 6.0, to produce weak alignment of the complex. The extent of alignment was checked by measuring the splitting of the deuterium resonance from D_2_O (∼ 8 Hz). One-bond HN RDCs were recorded using the in-phase/anti-phase pulse sequence [43], with 512 complex *t1* (^15^N) points for both the isotropic and anisotropic samples. The alignment tensor components were calculated by the PALES program [44]. All NMR spectra were processed and analyzed using NMRPipe package.

### ^15^N NMR Relaxation Measurements

^15^N R1, R2, and ^15^N-{^1^H} NOE experiments were performed on A13DBD at 285K and A13DBD•DNA at 310K using standard pulse sequences described previously [45]. Longitudinal magnetization decay was recorded using six different times: 0.00, 0.10, 0.25, 0.50, 1.00 and 2.00 s. The transverse magnetization decay was recorded with eight different delays: 0.000, 0.008, 0.016, 0.024, 0.032, 0.048, 0.064 and 0.080 s. To check the sample stability, the transverse magnetization decay at 0.016 s was verified unchanged before and after each set of measurements of both ^15^N R1 and R2 experiments. ^15^N-{^1^H} NOE values were obtained by recording two sets of spectra in the presence and absence of a 3 s proton saturation period. The NOE experiments were repeated 3 times to calculate the average and standard deviation of the NOE values. The overall rotational correlation time for backbone amide motion was determined using the protocol described previously [46].

### Structure Calculation

Backbone and side-chain NMR resonances were assigned as described previously [14], [23]. Analysis of NOESY data determined nearly 800 interproton distance relationships throughout the free protein [47]. The NMR-derived distances and dihedral angles then served as constraints (see Table 1) for calculating the three-dimensional structure of free protein using distance geometry and restrained molecular dynamics. Structure calculations were performed using the YASAP protocol within X-PLOR [48], [49], as described previously [50]. A total of 778 interproton distance constraints were obtained by analysis of ^13^C-edited and ^15^N-edited NOESY-HSQC spectra (120 ms mixing time) of ^13^C/^15^N-labeled A13DBD. In addition to the NOE-derived distance constraints, the following additional constraints were included in the structure calculation: 86 dihedral angle constraints (*φ* and ψ); 54 distance constraints for 27 hydrogen bonds verified by identifying slowly exchanging amide protons in hydrogen-deuterium exchange experiments. Fifty independent structures were calculated, and the 15 structures of lowest energy were selected. The average total and experimental distance energy were 1658 ± 19 and 125 kcal•mol^-1^. The average root-mean-square (rms) deviation from an idealized geometry for bonds and angles were 0.0089 Å and 1.96°. None of the distance and angle constraints were violated by more than 0.4 Å and 4°, respectively.

The NMR structure of A13DBD bound to an 11-mer DNA duplex (Fig. 1B) was calculated on the basis of intermolecular NOEs and residual dipolar couplings using Haddock 2.0 [51] (http://haddock.chem.uu.nl/). The starting structure of the DNA duplex (B-form) was generated using 3D-DART [52]. The B-form duplex structure in the complex was experimentally verified by observing characteristic NOE patterns from the DNA in the complex. The NMR-derived structure of free A13DBD determined above was used as an initial starting structure in the HADDOCK calculation. A few additional dihedral angle restraints (generated by TALOS) were also included to extend the length of helix 3 (residues 376–381), as determined by chemical shift index [23]. A total of 38 intermolecular NOE distance restraints from filtered NOESY experiments, including 25 unambiguous restraints and 13 ambiguous restraints (mainly from three residues in the N-terminal flexible loop, K322, R324 and Y327), were included in the HADDOCK calculation as well as conformational restraints for the DNA. The structure calculation protocol consists of three stages: rigid-body docking, semi-flexible simulated annealing, and refinement in explicit solvent as described previously [53]. After rigid-body docking, 200 lowest-energy structures were selected for semi-flexible refinement using all the NMR experimental restraints above. The protein side chains from residues that exhibit intermolecular NOEs with DNA were allowed to move in the semi-flexible annealing stage, and the N-terminal unstructured residues (G320 to T328) were set to remain flexible during the refinement. DNA bases (A4–A8 and T4*–T8*) were defined as active, and considered to be flexible during the semi-flexible annealing. The structures were further refined in an explicit solvent including all NMR derived restraints. In order to add the H_N_-N residual dipolar couplings into the structure refinement, the ensemble of 10 lowest energy structures generated from the first simulated annealing were used to calculate the axial and rhombic components of the alignment tensor (*D*_a_ and *D*_r_) using the PALES program [44]. The H_N_-N RDCs (total 25 ^1^ *D*_NH_ RDC values in the structurally rigid region) were introduced in the semi-flexible annealing and water refinement stages as direct restraints (using the SANI statement). Ten structures having the lowest HADDOCK energy were selected and went through another stage of refinement using all NMR experimental restraints. The ensemble of 10 final structures was superimposed with a root-mean-squared deviation of 0.67 Å (A13DBD) and 0.69 Å (DNA) (see Table 3 for structural statistics). A Ramachandran analysis of the ensemble of structures (evaluated by Procheck) revealed 84.4% of residues in the most favored regions, 14.1% in additional allowed regions, 1.5% in generously allowed regions, and 0% in disallowed regions. Thus, the NMR-derived structures of A13DBD bound to 11-mer DNA show good convergence and are well defined by the NMR restraints.

### Luciferase Assays

NG108-15 cells (ATCC#HB-12317) were maintained in DMEM media (Gibco) supplemented with 10% FBS (Atlanta Biologicals), HAT (Invitrogen) and 1% penicillin/streptomycin. Cells (1×10^5^) were seeded in 12-well plates and grown for 24 h at 37°C with 5% CO_2_. Transfections were performed using FuGENE6 transfection reagent (Roche), 0.1 µg pRL-CMV Renilla, and 0.25 µg pCAGGS-HOXA13 wild type or mutants, along with 0.5 µg of a pGL4.23 plasmid (Promega) containing an EphA7 *cis*-regtulatory element previously shown to be regulated by HOXA13 [22]. Empty pGL4.23 and pCAGGs expression vectors were used as controls. The Dual-Glo Luciferase Assay system (Promega) was used to detect luciferase activity 24 h post-transfection in OptiPlate-96F black plates using a Fusion Microplate Analyzer (Perkin Elmer). Six replicates of each transfection were performed and the transfection assay was repeated a total of 3 separate times. Results were normalized for transfection efficiency using a *Renilla* luciferase expression vector as described by the manufacturer (Promega). Because the pGL4.23 vector also contains several HOXA13 binding sites, background activation of the empty pGL4.23 by HOXA13 was also subtracted from the final luciferase levels after *Renilla* normalization.

### Fluorescence Anisotropy

Fluorescence polarization anisotropy was performed using a Beacon 2000 fluorescence polarization anisometer (Invitrogen). Self-annealing oligonucleotides were synthesized carrying a fluorescein via a hexyl linker (6-carboxyfluorescein) at the 5′ end and purified by high pressure liquid chromatography (Integrated DNA Technologies). Oligonucleotide sequences are presented in Table S1. Oligonucleotides were resuspended as a 100 µM stock in Tris-EDTA buffer, diluted to 10 µM in 20 mM Tris pH 7.5, 80 mM KCl, 10 mM MgCl_2_, 0.2 mM EDTA. The oligonucleotides were then denatured at 95°C for 10 min, and annealed by cooling to room temperature for 30 min. DNA binding affinity was determined using a fixed concentration of DNA (1 nM) and increasing concentrations of HOXA13 DNA binding domain (A13DBD). Wild type A13DBD protein (0–512 nM) or V373G (0–4.9 µM) were added to a solution containing 1 nM fluorescein-labeled DNA in 20 mM Tris pH 7.5, 80 mM KCl, 10 mM MgCl2, 0.2 mM EDTA, 1 mM dithiothreitol, and incubated at 15°C for 20 min. Measurements were collected at 15°C with a 10-s delay. The dissociation constants were calculated as previously described [8]. All results are based on three independent measurements for each protein and oligonuleotide combination. In the competitive displacement assays, increasing concentrations (0–5 µM) of unlabeled competitor DNA was added to either 400 nM wt A13 or 4 µM V373G protein bound to 1 nM labeled DNA. Measurements were collected at 15°C with a 10-s delay and 3 independent experiments were performed.

## Supporting Information

Figure S1 NMR titration of DNA binding to A13DBD. (**A**) ^1^H NMR spectra of duplex DNA with stepwise addition of A13DBD (molar ratio indicated on the right side) in 20mM phosphate buffer (pH 6.0) with 80mM KCl, 5mM MgCl_2_ and 10%D_2_O at 285K. (**B**) ^1^H NMR spectrum of A13DBD/duplex DNA complex in 20mM phosphate buffer (pH 6.0) with 80mM KCl, 5mM MgCl_2_ and 10%D_2_O at 310K. Spectral assignments of DNA imino resonances are shown.
(DOC)

Figure S2 Molar mass of A13DBD/DNA complex determined by ^15^N-NMR relaxation data. Spin-lattice relaxation rate constants (A) and spin-spin relaxation rate constants (B) are plotted versus residue number. All data were determined at 600MHz ^1^H frequency and 310K. Error bars are given as the standard deviation of three independent measurements.
(DOC)

Figure S3 Differential scanning calorimetry thermograms of A13DBD and mutants (see Methods).
(DOC)

Figure S4 Overlay of two-dimensional ^1^H-^15^N HSQC spectra of A13DBD (black) and mutants I366 (blue), N370 (cyan) and V373 (red) at 285K.
(DOC)

Figure S5 Luciferase assays in NG108-15 cells using a series of HOXA13 mutants. Percent luciferase activities (relative to wt control) are indicated on the *y* axis, and the various pCAGGS-HOXA13 mutants are plotted on the *x* axis. Values represent the mean percent luciferase activity from three independent experiments. Error bars represent the standard error for the three independent experiments.
(DOC)

Figure S6 Size-exclusion chromatography profiles of A13DBD and mutants (F344A (red) and F344A/R337G (blue)) in complex with duplex DNA.
(DOC)

Figure S7 Isothermal calorimetric titration monitoring A13DBD binding to 11-mer DNA duplex as described in the text. Representative ITC data are shown for wildtype (A) and N370A (B) A13DBD.
(DOC)

Figure S8 NMR spectral analysis and assignment of DNA resonances. (A) The superposition of 2D ^13^C-filtered (F1 and F2) NOESY spectrum of ^13^C/^15^N-labeled A13DBD bound to unlabeled duplex DNA (wildtype, black) and three T-to-dU mutants, T5U (purple), T17U (red) and T19U (green), recorded in 99.9% D_2_O at pH 6.0. The ^1^H chemical shift of three pyrimidine methyl groups (from T5, T17, T19) can be assigned unambiguously as shown in the spectra based on T-to-dU mutants. (B) Sequential NOE assignments from 2D ^13^C-filtered NOESY of ^13^C/^15^N-labeled A13DBD bound to unlabeled duplex DNA, recorded in 99.9% D_2_O, showing the sequential NOE connections between methyl protons of T_i_ and H6 of T_i-1_ from T19 to T21 and T14 to T17.
(DOC)

Table S1 Oligonucleotide sequences used in fluorescence anisotropy assays.
(DOC)

Movie S1 (WMV)
