# Supplementary figures and images for: Structural Basis for Sequence Specific DNA Binding and Protein Dimerization of HOXA13
Source: PLoS One. 2011 Aug 1;6(8):e23069. doi: 10.1371/journal.pone.0023069 (PMC3148250; doi:10.1371/journal.pone.0023069)

**Figure S1.**


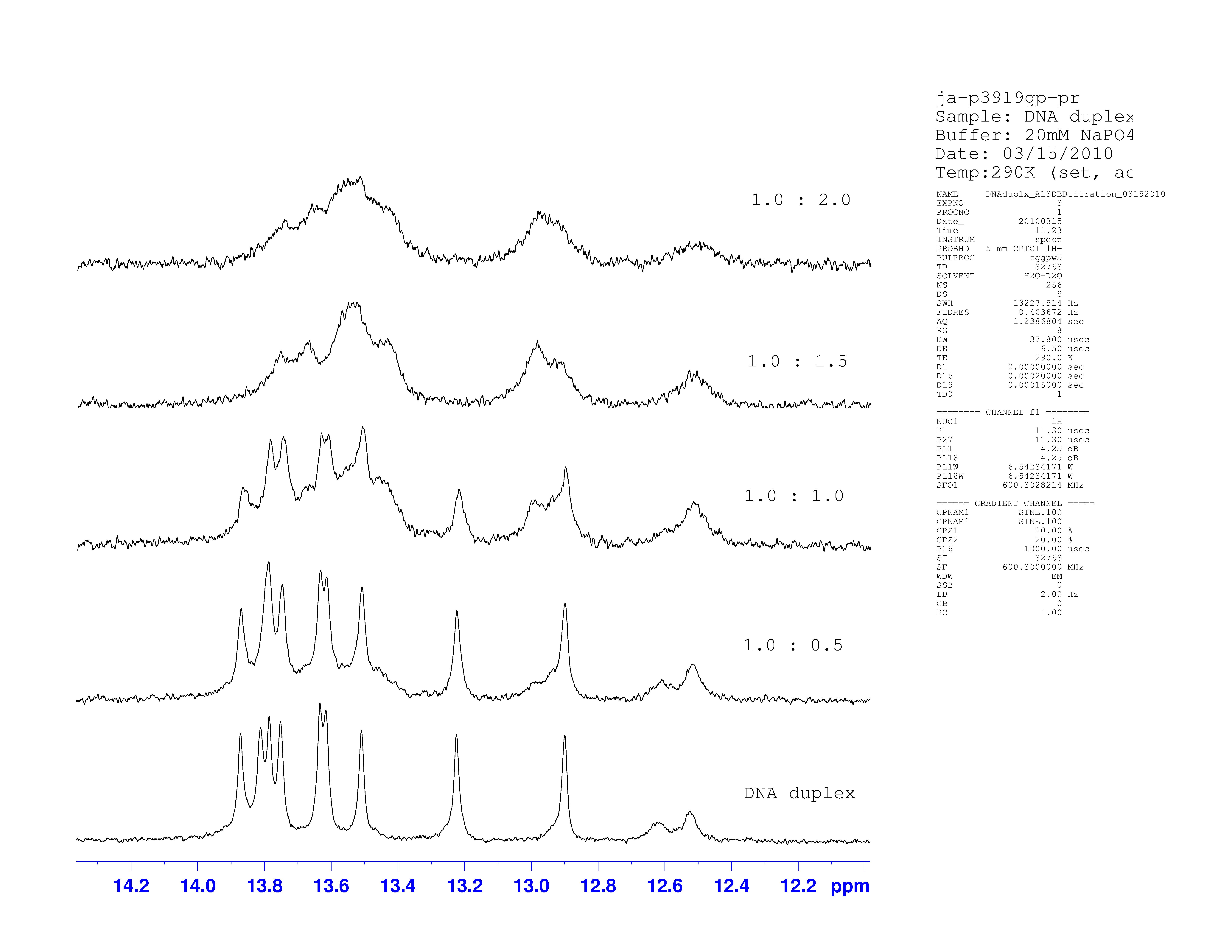


Free DNA

0.3

0.6

1.0

1.5

T5

T6*

T4*

T9* T10

T2* T7* T3*

T8*

**A**

G11* G1*

A13DBD/DNA at 285K

**B**


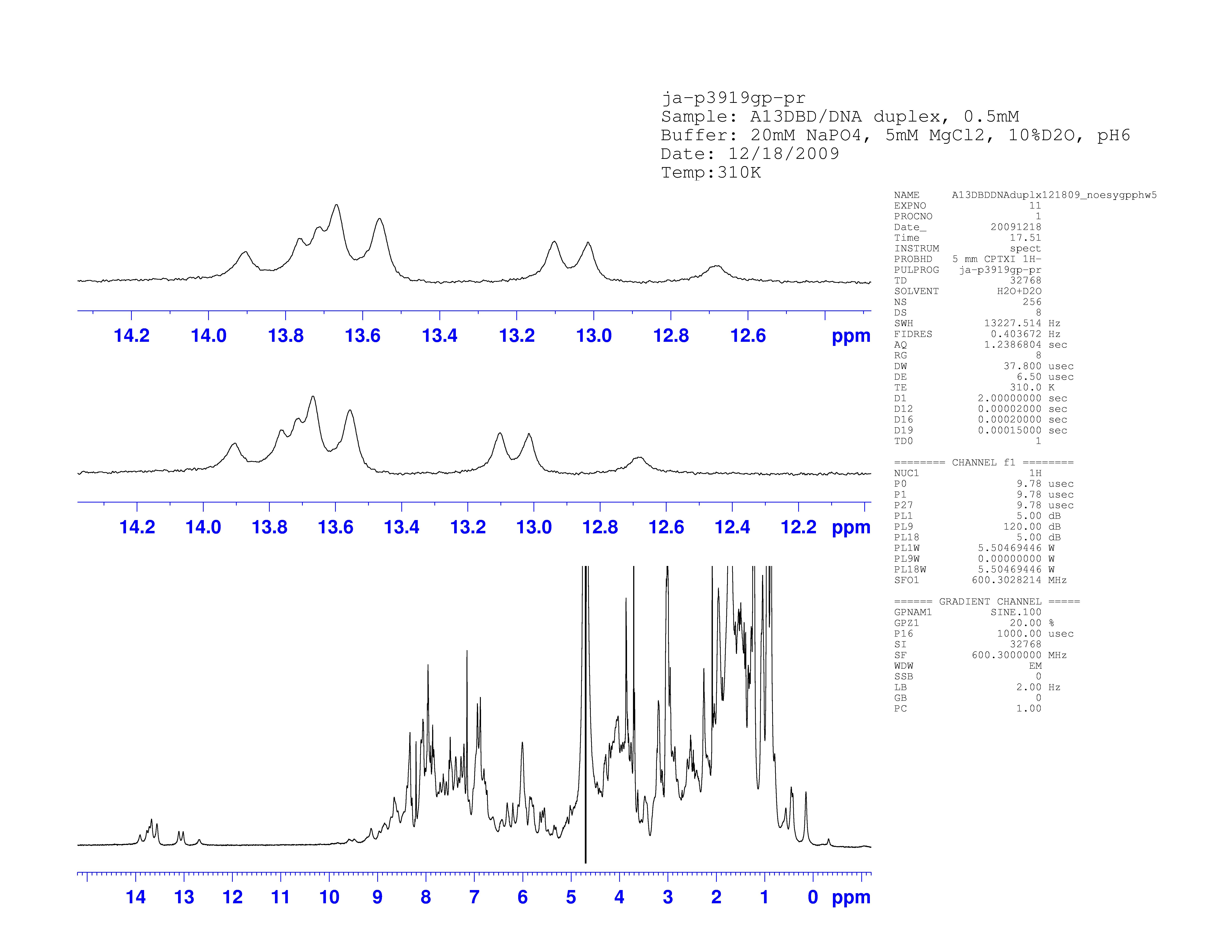


T4*

T3*

T7*

T6*

T5

T2*

T8*

T10

G11*/G1*

T9*

A13DBD/DNA at 310K

Supplement: Figure S1 — NMR titration of DNA binding to A13DBD. (A) 1H NMR spectra of duplex DNA with stepwise addition of A13DBD (molar ratio indicated on the right side) in 20mM phosphate buffer (pH 6.0) with 80mM KCl, 5mM MgCl2 and 10%D2O at 285K. (B) 1H NMR spectrum of A13DBD/duplex DNA complex in 20mM phosphate buffer (pH 6.0) with 80mM KCl, 5mM MgCl2 and 10%D2O at 310K. Spectral assignments of DNA imino resonances are shown. (DOC) [file pone.0023069.s001.doc]

**Figure S3.** The VP-DSC thermograms of A13DBD and mutants (see Methods).


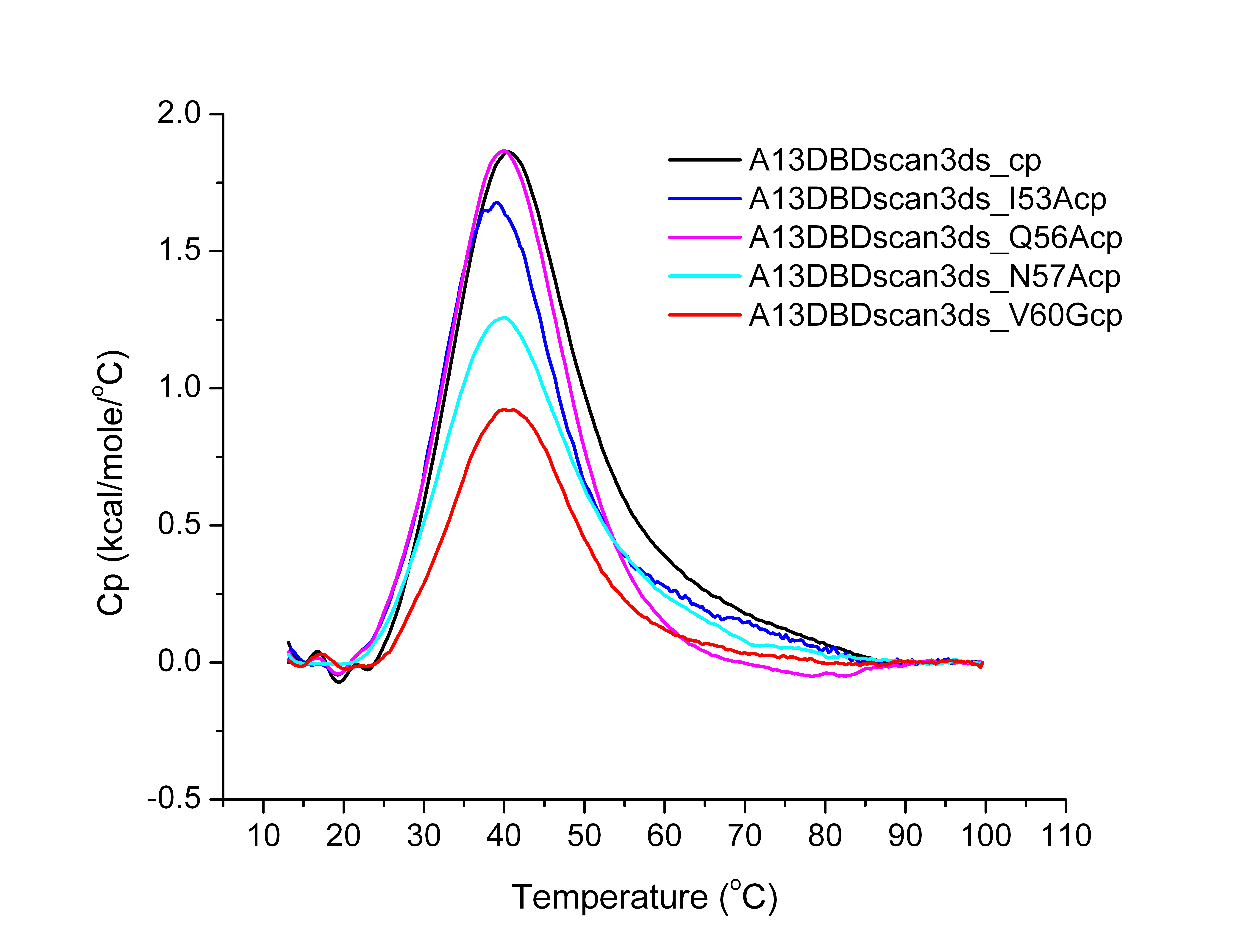

Supplement: Figure S3 — Differential scanning calorimetry thermograms of A13DBD and mutants (see Methods). (DOC) [file pone.0023069.s003.doc]

**Figure S4.**


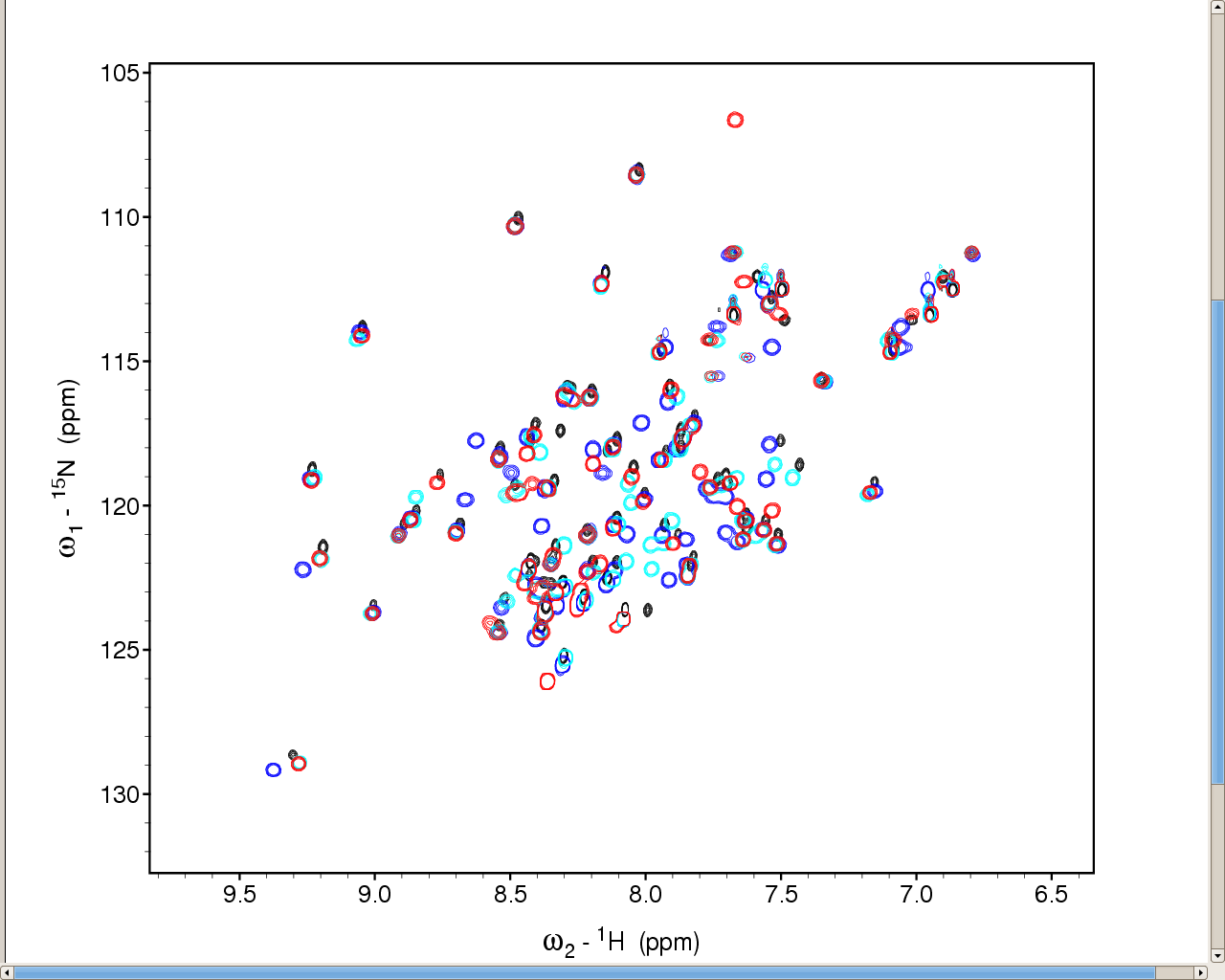

Supplement: Figure S4 — Overlay of two-dimensional 1H-15N HSQC spectra of A13DBD (black) and mutants I366 (blue), N370 (cyan) and V373 (red) at 285K. (DOC) [file pone.0023069.s004.doc]

**Figure S5.**


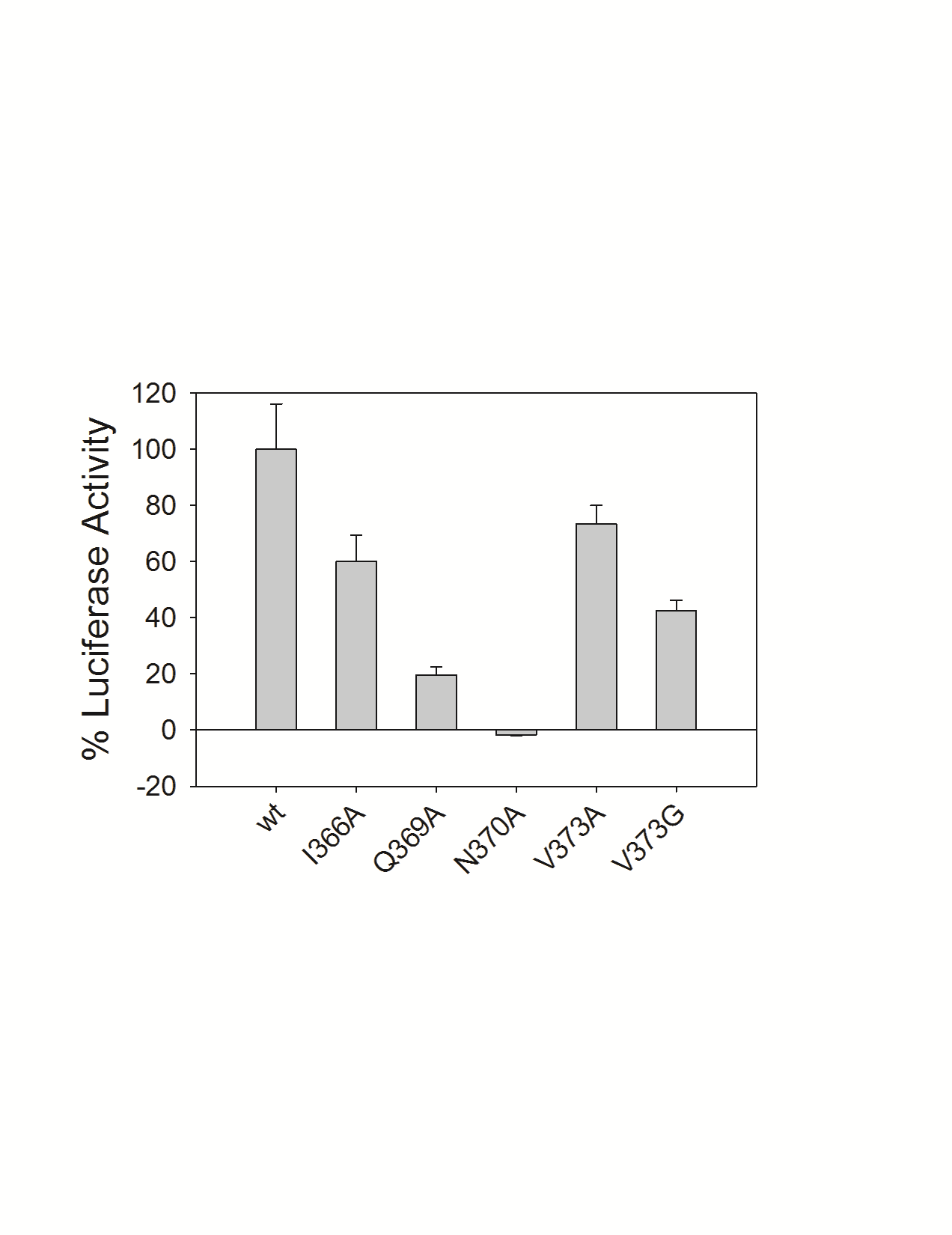

Supplement: Figure S5 — Luciferase assays in NG108-15 cells using a series of HOXA13 mutants. Percent luciferase activities (relative to wt control) are indicated on the y axis, and the various pCAGGS-HOXA13 mutants are plotted on the x axis. Values represent the mean percent luciferase activity from three independent experiments. Error bars represent the standard error for the three independent experiments. (DOC) [file pone.0023069.s005.doc]

**Figure S7**

A B


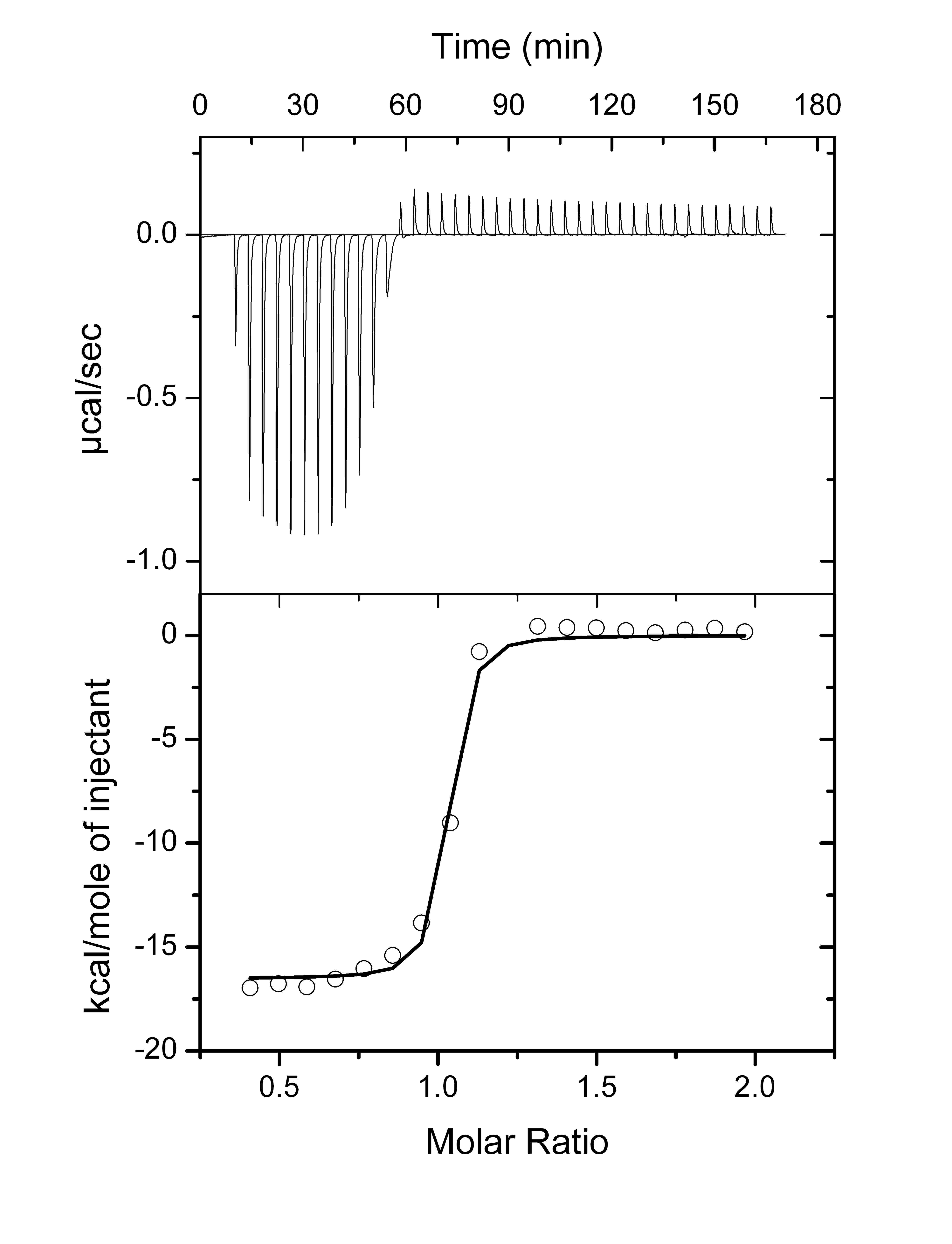


A


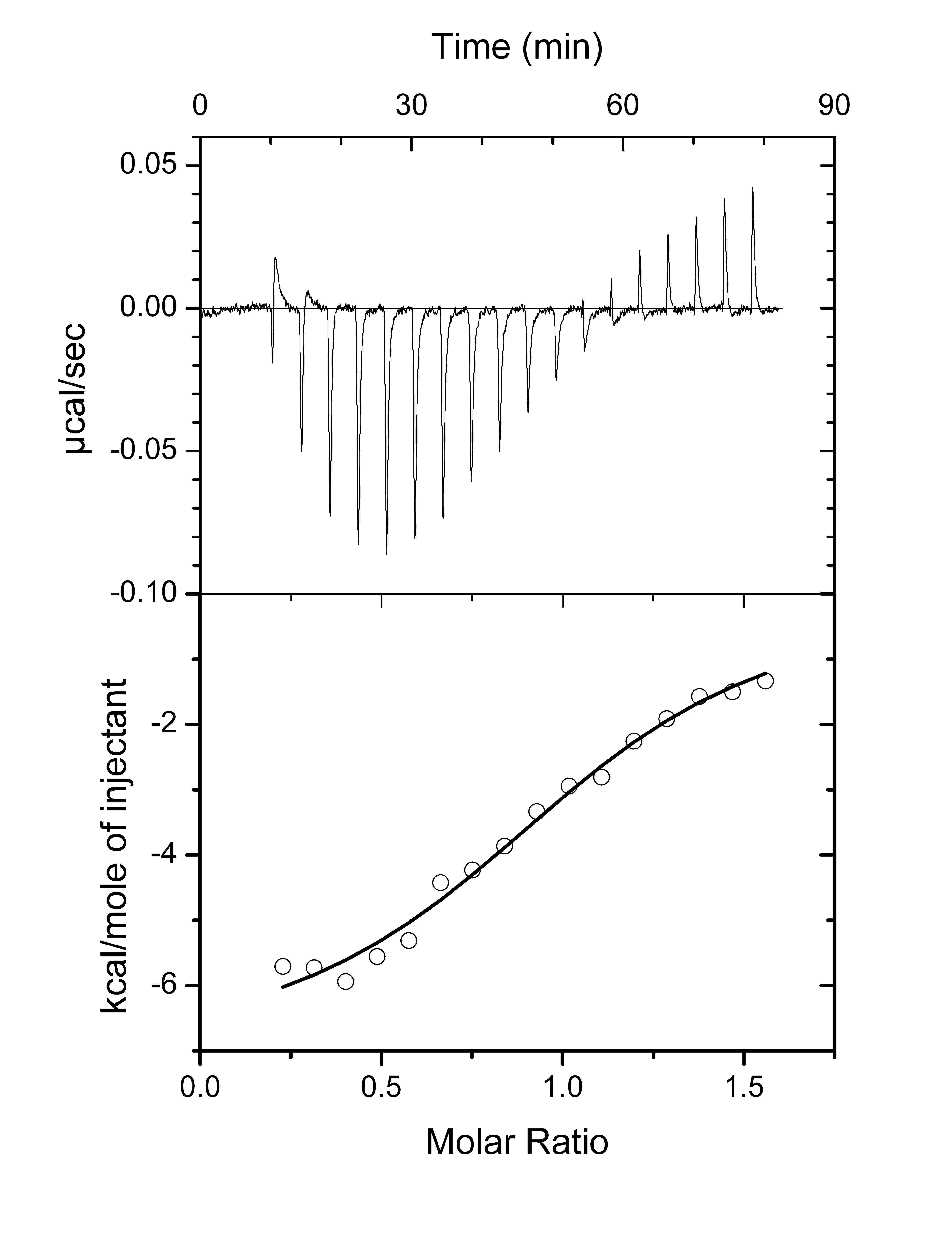


B

Supplement: Figure S7 — Isothermal calorimetric titration monitoring A13DBD binding to 11-mer DNA duplex as described in the text. Representative ITC data are shown for wildtype (A) and N370A (B) A13DBD. (DOC) [file pone.0023069.s007.doc]

**Figure S8.**


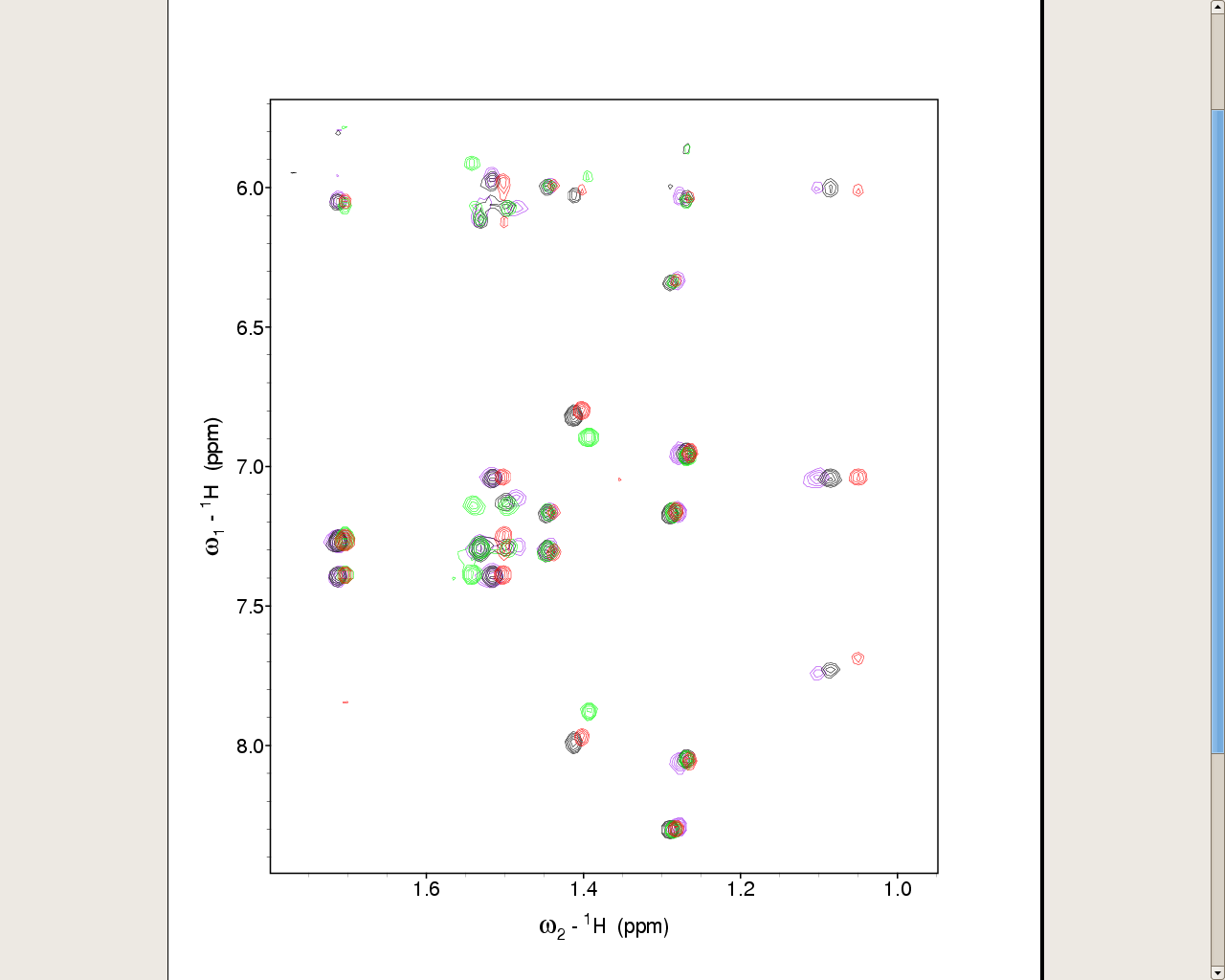


CH3-T4* = 1.08

CH3-T5 = 1.41

CH3-T6* = 1.50

A


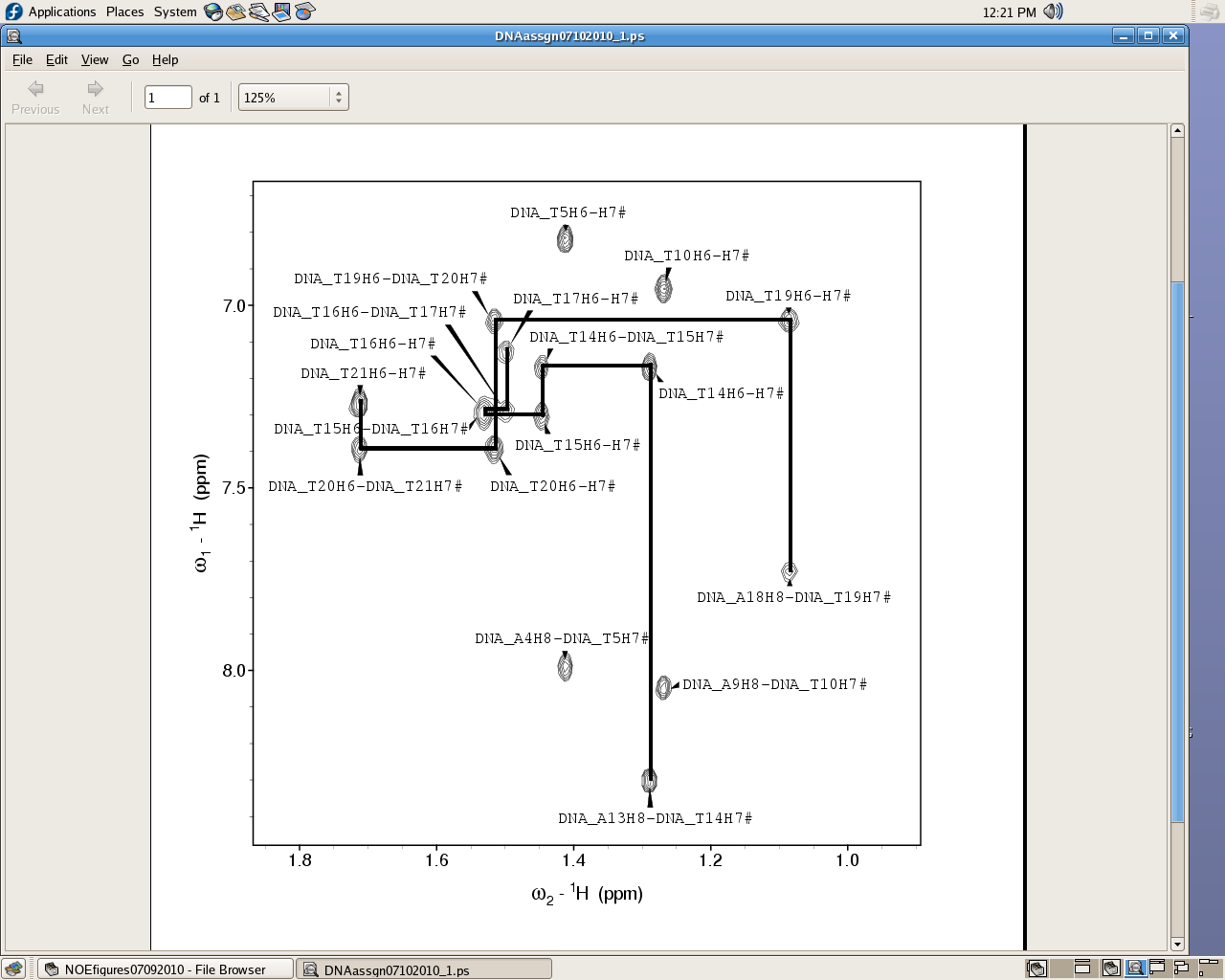


B

Supplement: Figure S8 — NMR spectral analysis and assignment of DNA resonances. (A) The superposition of 2D 13C-filtered (F1 and F2) NOESY spectrum of 13C/15N-labeled A13DBD bound to unlabeled duplex DNA (wildtype, black) and three T-to-dU mutants, T5U (purple), T17U (red) and T19U (green), recorded in 99.9% D2O at pH 6.0. The 1H chemical shift of three pyrimidine methyl groups (from T5, T17, T19) can be assigned unambiguously as shown in the spectra based on T-to-dU mutants. (B) Sequential NOE assignments from 2D 13C-filtered NOESY of 13C/15N-labeled A13DBD bound to unlabeled duplex DNA, recorded in 99.9% D2O, showing the sequential NOE connections between methyl protons of Ti and H6 of Ti-1 from T19 to T21 and T14 to T17. (DOC) [file pone.0023069.s008.doc]
