# Supplementary material for: Structural Basis for Sequence Specific DNA Binding and Protein Dimerization of HOXA13
Source: PLoS One. 2011 Aug 1;6(8):e23069. doi: 10.1371/journal.pone.0023069 (PMC3148250; doi:10.1371/journal.pone.0023069)
Supplement: Figure S2 — Molar mass of A13DBD/DNA complex determined by 15N-NMR relaxation data. Spin-lattice relaxation rate constants (A) and spin-spin relaxation rate constants (B) are plotted versus residue number. All data were determined at 600MHz 1H frequency and 310K. Error bars are given as the standard deviation of three independent measurements. (DOC) [file pone.0023069.s002.doc]

**Figure S2**

A

B
