# Supplementary material for: Structural Basis for Sequence Specific DNA Binding and Protein Dimerization of HOXA13
Source: PLoS One. 2011 Aug 1;6(8):e23069. doi: 10.1371/journal.pone.0023069 (PMC3148250; doi:10.1371/journal.pone.0023069)
Supplement: Figure S6 — Size-exclusion chromatography profiles of A13DBD and mutants (F344A (red) and F344A/R337G (blue)) in complex with duplex DNA. (DOC) [file pone.0023069.s006.doc]

**Figure S6.**

A13DBD/DNA

F31A A13DBD/DNA

F31A&R24G A13DBD/DNA

Duplex DNA
