# Supplementary material for: Structural Basis for Sequence Specific DNA Binding and Protein Dimerization of HOXA13
Source: PLoS One. 2011 Aug 1;6(8):e23069. doi: 10.1371/journal.pone.0023069 (PMC3148250; doi:10.1371/journal.pone.0023069)
Supplement: Table S1 — Oligonucleotide sequences used in fluorescence anisotropy assays. (DOC) [file pone.0023069.s009.doc]

**Table S1.** Oligonucleotide sequences used in fluorescence anisotropy assays.

Consensus Seq.:CAAATAAAATCCCCCATTTTATTTG

T5C: CAAACAAAATCCCCCATTTTGTTTG

T5U: CAAAUAAATCCCCCATTTTATTGTG

T6*C: CAAATGAAATCCCCCATTTCATTG

T6*U: CAAATAAAATCCCCCATTTUATTG

Scrambled: TGACTGACTGACTGCCCCCCAGTCAG
